# Safe regions of miniscrew implantation for distalization of mandibular dentition with CBCT

**DOI:** 10.1186/s40510-019-0297-6

**Published:** 2019-12-09

**Authors:** Haibo Liu, Xiaoxue Wu, Jun Tan, Xiao Li

**Affiliations:** 1 Department of Stomatology, General Hospital of Southern Theater Command of the Chinese People’s Liberation Army (Guangzhou Liuhuaqiao Hospital), No.111, Liuhua road, Guangzhou, 510016 Guangdong China; 2 Department of Orthodontics, Key Laboratory of Oral Medicine, Guangzhou Institute of Oral Disease, Stomatology Hospital of Guangzhou Medical University, Guangzhou, 510140 Guangdong China

**Keywords:** Mandibular buccal shelf, Cone-beam computed tomography, Miniscrew, Mandibular dentition

## Abstract

**Background:**

To assess the anatomy of the mandibular buccal shelf (MBS) with cone-beam computed tomography (CBCT) and to identify the region of miniscrew implantation for the distalization of mandibular dentition.

**Materials and methods:**

The MBS was assessed in 80 patients at four regions as follows: (i) between the buccal root of the mandibular second premolar and the mesiobuccal root of the first molar (L5_b_–L6_mb_), (ii) between the mesiodistal root of the first molar (L6_mb_–L6_db_), (iii) between the distobuccal root of the first molar and the mesiobuccal root of the second molar (L6_db_–L7_mb_), and (iv) between the mesiodistal roots of the second molar (L7_mb_–L7_db_). The buccal alveolar bone thickness, the narrowest inter-radicular space at the buccal side of the roots, and the distance between the implantation site and the mandibular neural tube were measured at horizontal planes of 3, 5, 7, and 9 mm from the alveolar crest.

**Results:**

The buccal alveolar bone thickness increased from the premolar to the molar and from the crest edge to the mandibular roots. The L7_mb_–L7_db_ region had the thickest buccal alveolar bone of 7.61 mm at a plane of 9 mm. The buccal inter-radicular spaces were smallest in the L7_mb_–L7_db_ region and greatest in the L6_db_–L7_mb_ region. The distances from the implantation site to the mandibular neural tube at planes of 3, 5, 7, and 9 mm were all > 13 mm from the L6 region to the L7 region.

**Conclusions:**

The L6_db_–L7_mb_ region should be the first choice for miniscrew implantation in the MBS for the distalization of mandibular dentition.

## Background

To correct tooth movement, miniscrews are routinely implanted in various positions in the maxilla and mandible, including the inter-radicular space, infrazygomatic crest, paramedian palate, and retromolar area [1–3]. Inter-radicular miniscrews are most commonly used, but their insertion is often problematic in the posterior mandible [4]. Recently, the mandibular buccal shelf (MBS) has been used as the insertion site for orthodontic miniscrews, as this region offers sufficient bone and adequate bone quality for miniscrew insertion [5].

**Fig. 1:**
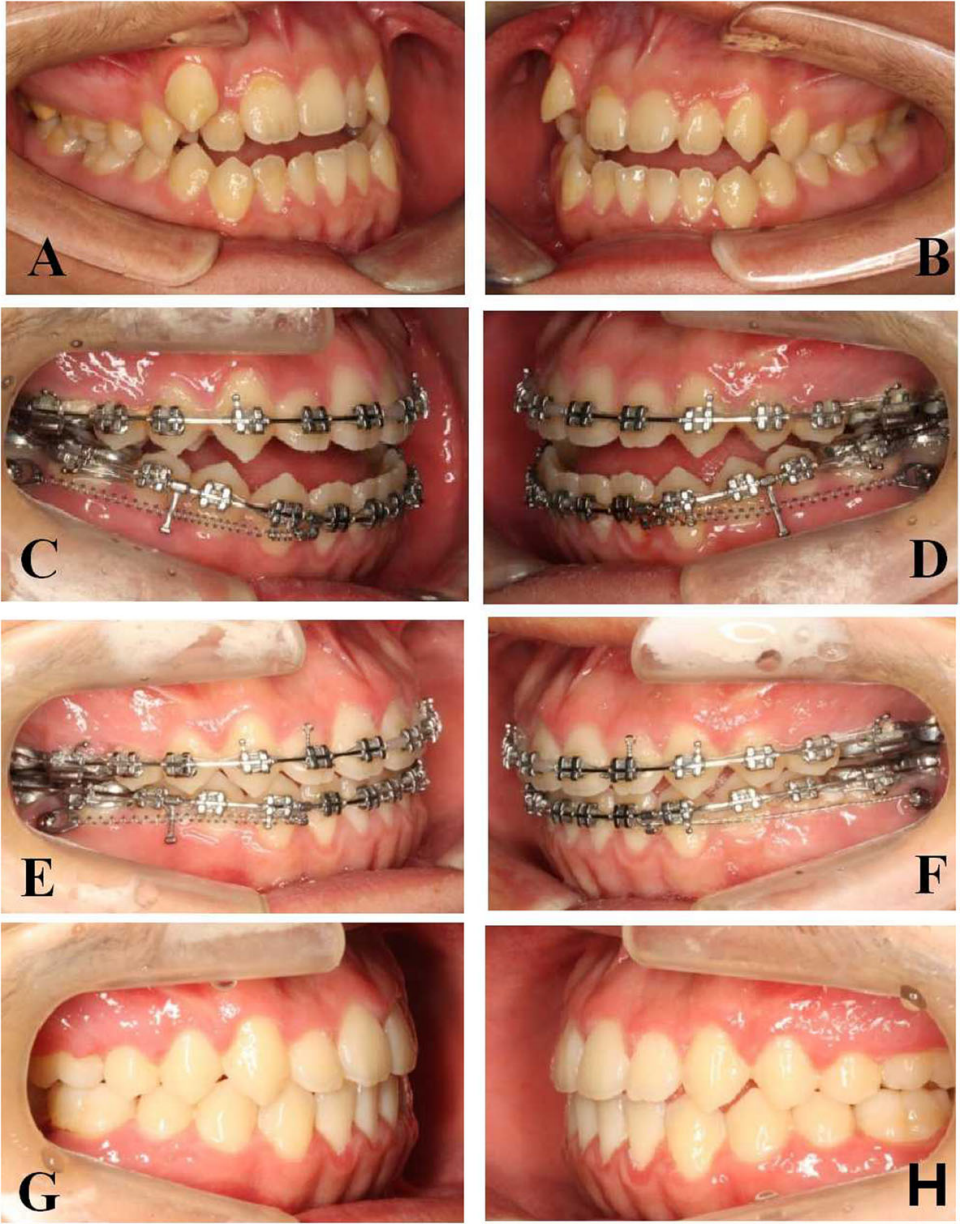
Images for distalization of the entire mandibular dentition with miniscrews inserted in the MBS region. **a**, **b** Intraoral images before treatment. **c**–**f** Miniscrew insertion for the distalization of mandibular dentition. **g**, **h** Intraoral images after treatment

Distal en masse movement of mandibular dentitions is quite effective in patients with class III malocclusions. Prior to the introduction of the miniscrew in the field of orthodontics, it was difficult to move mandibular dentitions. With the aid of a miniscrew inserted in the MBS region, mandibular dentitions can be successfully moved [6–8] (Fig. 1).

The MBS includes the proximal area of the first molar, which is between the first and second molars, as well as the distal area of the second molar. The MBS offers improved stability, and it associates with a lower failure rate for miniscrew insertion [9]. However, there are various anatomical differences in this region. Unfortunately, few studies have examined the anatomy of the MBS. The objective of this study was to analyze the buccal bone thickness and buccal inter-radicular distance, as well as the relationship between the miniscrew and the inferior alveolar nerve in order to determine the most suitable MBS sites for miniscrew insertion.

## Materials and methods

### Samples

Cone-beam computed tomography (CBCT) images of 80 patients (30 males and 50 females; average age26 ± 7 years) were obtained from the Department of Orthodontics, Stomatology Hospital of Guangzhou Medical University. The CBCT images were collected from October 2015 to January 2018 and fulfilled the following criteria: patients aged 18 to 37 years with full permanent dentition and evidence of fully erupted mandibular second molars (except third molars), no impacted teeth, no history of previous orthodontic treatment, and no severe crowding were included, whereas those with periodontal disease and craniofacial anomalies or systemic diseases were excluded.

### Methods

**Fig. 2:**
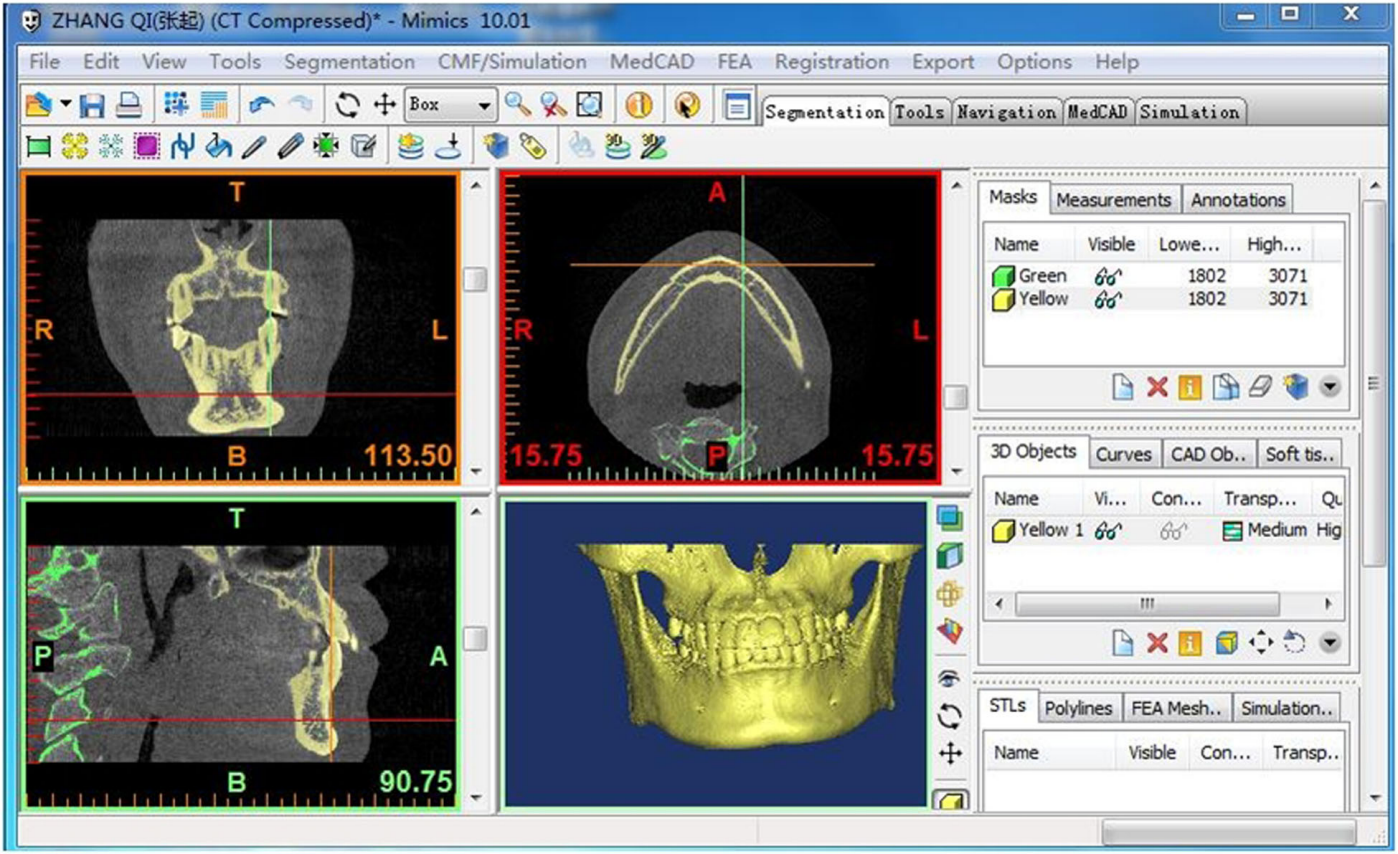
CBCT images of three slices and 3D reconstruction by Mimics

Eighty CBCT images were obtained with a CBCT instrument (NewTom, Verona, Italy) at 110 kV and 0.07 mA. The CBCT images were formatted into standard DICOM images and reconstructed into continuous slices of 0.3 mm in thickness. All images were analyzed by Mimics (version 10.0; Materialise, Leuven, Belgium) (Fig. 2).

To assess the safe regions of the miniscrews implanted in the MBS for the distalization of mandibular dentition, four sites were measured in the buccal shelf on each side as follows: (i) between the buccal root of the mandibular second premolar and the mesiobuccal root of the first molar (L5_b_–L6_mb_), (ii) between the mesiodistal roots of the first molar (L6_mb_–L6_db_), (iii) between the distobuccal root of the first molar and the mesiobuccal root of the second molar (L6_db_–L7_mb_), and (iv) between the mesiodistal roots of the second molar (L7_mb_–L7_db_). The buccal alveolar bone thickness, the narrowest inter-radicular space at the buccal side of the roots, and the distance between the implantation site and the mandibular neural tube were measured.

**Fig. 3:**
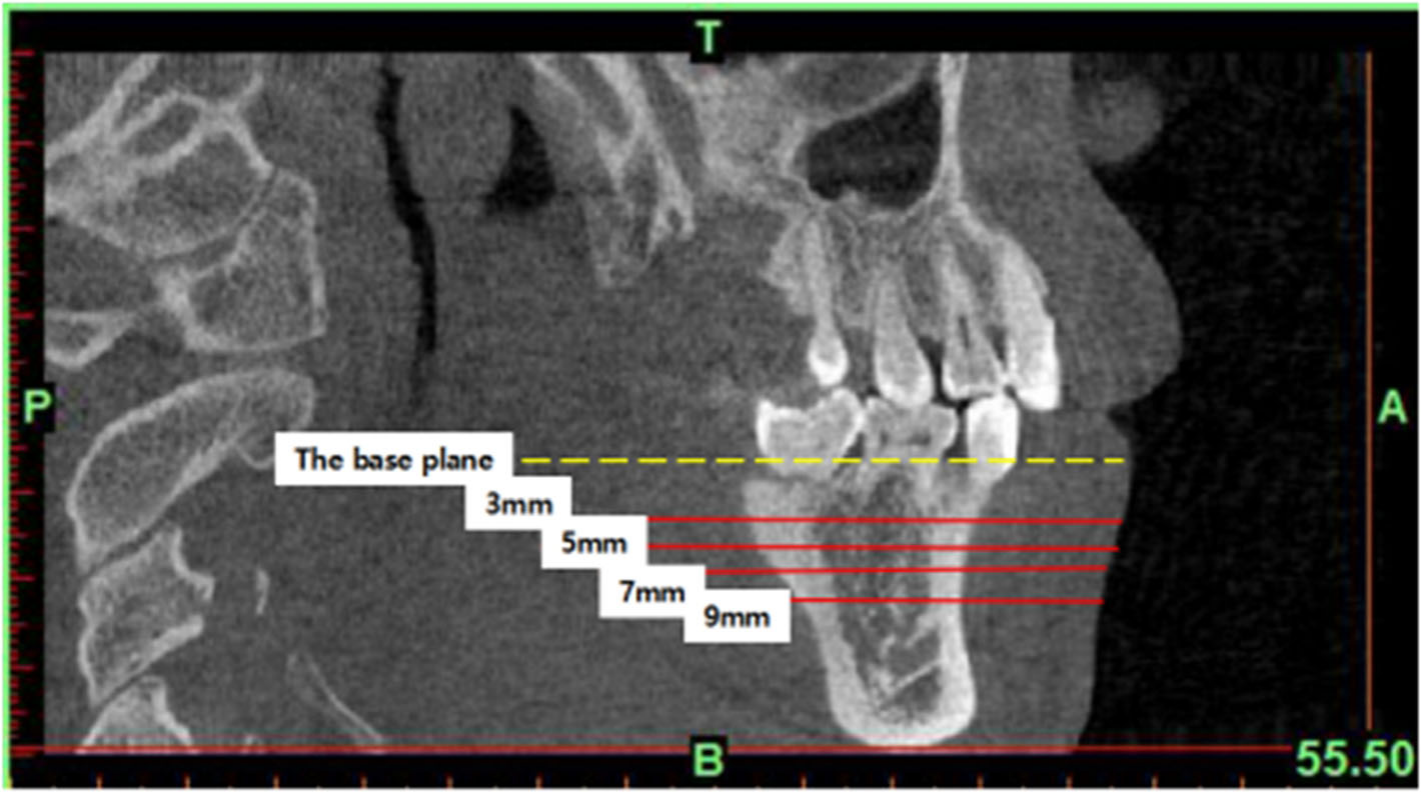
Reference lines for planes of 3, 5, 7, and 9 mm below the measurement base plane (alveolar crest edge) in the sagittal view

**Fig. 4:**
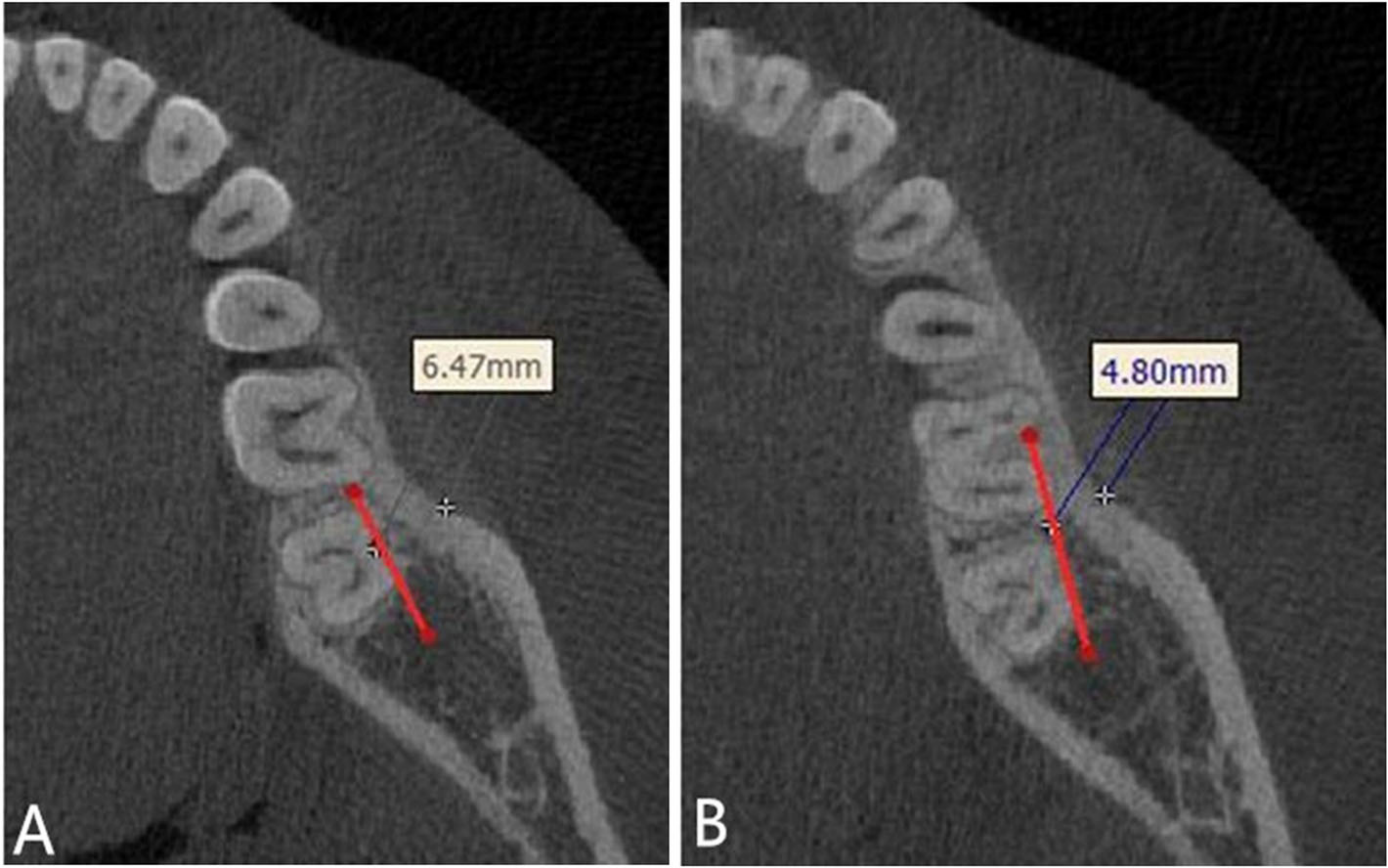
Measurement of buccal alveolar bone thickness (right-hand side). **a** A thickness of 5 mm from the alveolar crest between the mesiodistal roots of the second molar (L7_mb_–L7_db_). **b** A thickness of 7 mm from the alveolar crest between the first and second molar (L6_db_–L7_mb_)

For measuring the buccal alveolar bone thickness, the patient’s mandible was oriented in all three spatial planes. First, the base plane was determined, the highest point of the right and left alveolar crest in the first molar and the highest point of the right alveolar crest in the second molar were identified, the base plane was reoriented in order to pass through these three highest points. Second, sagittal and coronal images were adjusted so that the base plane was parallel to the frame’s border. The mid-sagittal plane was centered on the axial slice. The buccal alveolar bone thicknesses of these regions were measured at horizontal planes of 3, 5, 7, and 9 mm from the alveolar crest, parallel to the base plane (Figs. 3 and 4).

**Fig. 5:**
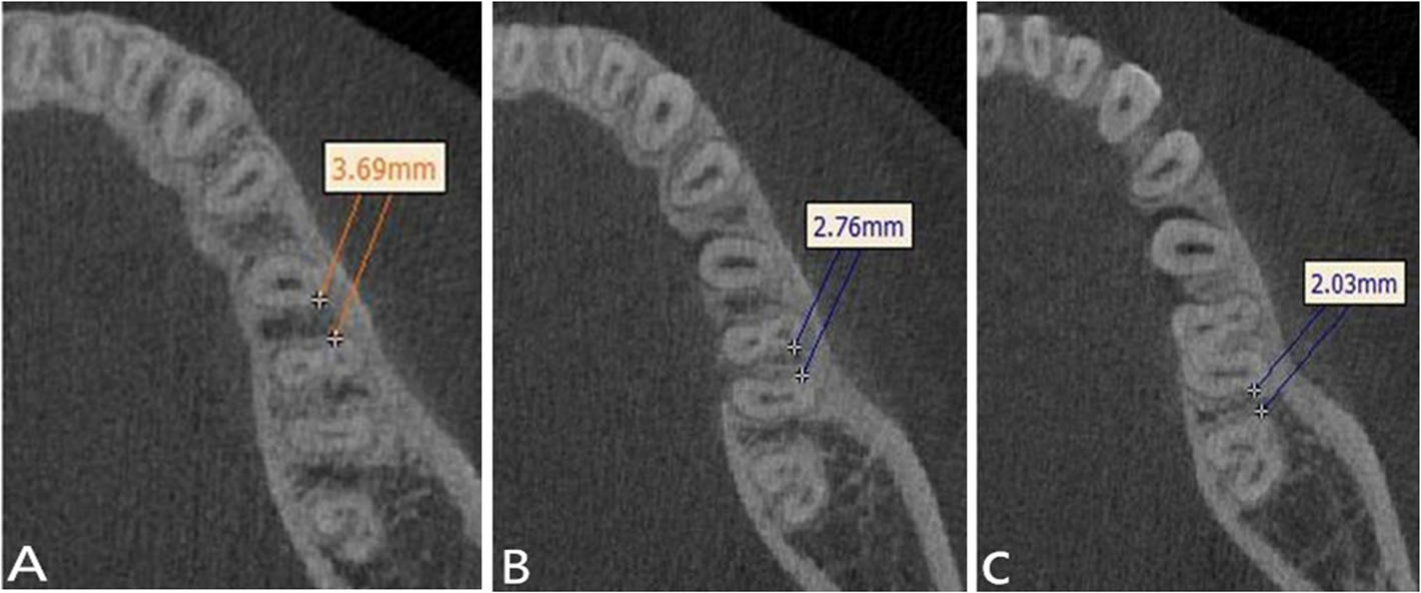
Measurement of the narrowest inter-radicular space at the buccal side of the roots (right-hand side). **a** A space of 7 mm from the alveolar crest between the second premolar and the first molar (L5_b_–L6_mb_). **b** A space of 7 mm from the alveolar crest between the mesiodistal roots of the first molar (L6_mb_–L6_db_). **c** A space of 7 mm from the alveolar crest between the first and second molar (L6_db_–L7_mb_)

The narrowest inter-radicular space at the buccal side of the roots was measured at L5_b_–L6_mb_, L6_mb_–L6_db_, L6_db_–L7_mb_, and L7_mb_–L7_db_ regions at planes of 3, 5, 7, and 9 mm from the buccal alveolar crest edge (Figs. 3 and 5).

The distance between the implantation site and the mandibular neural tube was measured at L6_mb_–L6_db_ or L7_mb_–L7_db_ regions at the coronal plane which was exactly through the midpoint of the mesial and distal direction of the first or second molar.

**Fig. 6:**
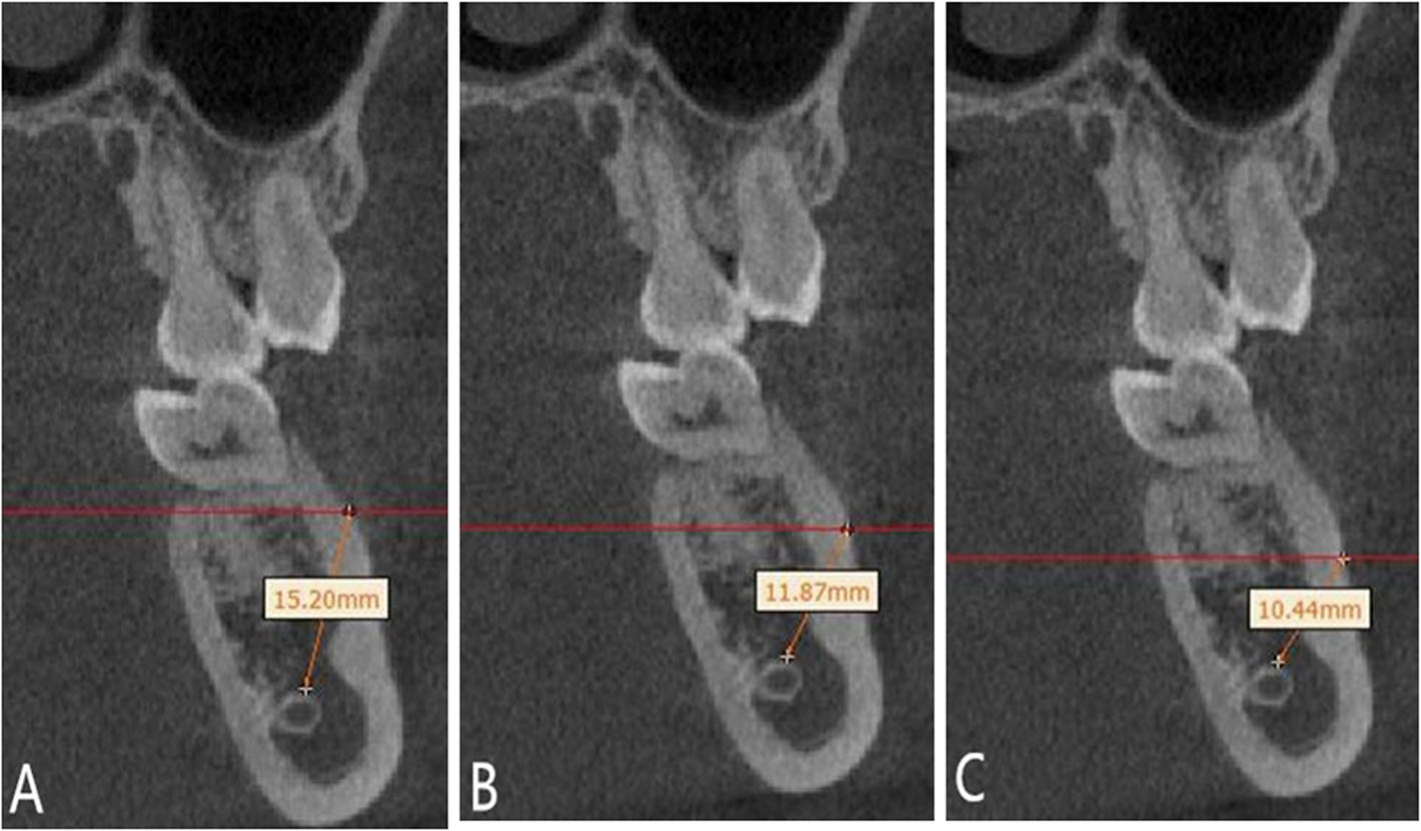
Measurement of the distance between the implantation site and the mandibular neural tube. **a** The implantation site at the plane of 5 mm from the alveolar crest and between the mesiodistal roots of the first molar (L6_mb_–L6_db_). **b** The implantation site at the plane of 7 mm from the alveolar crest and between the mesiodistal roots of the first molar (L6_mb_–L6_db_). **c** The implantation site at the plane of 9 mm from the alveolar crest and between the mesiodistal roots of the first molar (L6_mb_–L6_db_)

Miniscrews were implanted at planes of 3, 5, 7, and 9 mm from the alveolar crest at the coronal plane, and then the distance from the implantation site to the mandibular neural tube was measured (Fig. 6).

### Statistical analysis

SPSS 17.0 (Chicago, IL, USA) was used for statistical analysis. *P* values < 0.05 were considered significant. Measurements and data analysis were performed by the same investigator. Ten out of 80 volumetric tomographic images were randomly selected and repeated twice at an interval of 2 weeks by the same investigator. No statistical difference was found between the repeated measurements. Intraclass correlations showed high reliability (*r* = 0.8). A paired Student’s *t* test was used to test for differences between the left and right sides. No statistically significant differences were found (*P* > 0.05). The Kruskal–Wallis *H* test was applied to evaluate the differences in the buccal alveolar bone thickness, the narrowest inter-radicular space, and the distance from the implantation site to the mandibular neural tube in each measured region in each slice.

## Results

The buccal alveolar bone thicknesses at planes of 3, 5, 7, and 9 mm at four regions are shown in Table 1. There were significant differences in buccal alveolar bone thickness among L5_b_–L6_mb_, L6_mb_–L6_db_, L6_db_–L7_mb_, and L7_mb_–L7_db_ regions within the same plane (*P* < 0.05). The buccal alveolar bone thickness increased from the premolar to the molar. Although no significant difference was observed among different adjacent planes within the same region (*P* > 0.05), there were significant differences at the nonadjacent planes within the same region (*P* < 0.05). The buccal alveolar bone thickness increased from the crest edge to the mandibular root. The L5_b_–L6_mb_ region had the thinnest buccal alveolar bone of 1.02 mm at the plane of 3 mm, and the L7_mb_–L7_db_ region had the thickest buccal alveolar bone of 7.61 mm at the plane of 9 mm.

**Table 1:** Buccal alveolar bone thicknesses (in millimeters: mean ± SD) at the planes of 3, 5, 7, and 9 mm

	3 mm	5 mm	7 mm	9 mm
L5_b_–L6_mb_	1.02	1.36	2.51	3.93
SD	0.37	0.46	0.88	0.63
L6_mb_–L6_db_	1.80	2.15	3.29	4.86
SD	0.43	0.58	0.85	1.17
L6_db_–L7_mb_	2.88	3.45	5.07	6.39
SD	0.55	0.71	0.92	1.24
L7_mb_–L7_db_	3.86	4.92	6.17	7.61
SD	0.51	0.96	1.25	1.01

*L5*_*b*_ buccal root of the second premolar, *L6*_*mb*_ mesiobuccal root of the first molar, *L6*_*db*_ distobuccal root of the first molar, *L7*_*mb*_ mesiobuccal root of the second molar, *L7*_*db*_ distobuccal root of the second molar

The narrowest buccal inter-radicular spaces at planes of 3, 5, 7, and 9 mm at four sites are shown in Table 2. There were significant differences in the narrowest buccal spaces between the roots of different teeth and between the roots of the same tooth (*P* < 0.05). The inter-radicular space was significantly greater in L5_b_–L6_mb_ and L6_db_–L7_mb_ regions than that in L6_mb_–L6_db_ and L7_mb_–L7_db_ regions at the same plane. The narrowest buccal inter-radicular space increased from the crest edge to the mandibular root within the same region. The buccal inter-radicular spaces were the narrowest in the L7_mb_–L7_db_ region and the widest in the L6_db_–L7_mb_ region. The L6_db_–L7_mb_ region had the narrowest buccal inter-radicular space of > 3.5 mm at planes of 7 and 9 mm.

**Table 2:** Buccal narrowest inter-radicular space (in millimeters: mean ± SD) at the planes of 3, 5, 7, and 9 mm

	3 mm	5 mm	7 mm	9 mm
L5_b_–L6_mb_	1.94	2.69	3.31	3.84
SD	0.74	0.82	0.91	1.19
L6_mb_–L6_db_	0.99	1.75	2.29	2.54
SD	0.53	0.56	0.67	1.03
L6_db_–L7_mb_	2.78	3.22	3.92	4.97
SD	1.04	1.54	1.94	2.36
L7_mb_–L7_db_	0.79	1.30	1.39	2.04
SD	0.46	0.77	0.87	0.88

*L5*_*b*_ buccal root of the second premolar, *L6*_*mb*_ mesiobuccal root of the first molar, *L6*_*db*_ distobuccal root of the first molar, *L7*_*mb*_ mesiobuccal root of the second molar, *L7*_*db*_ distobuccal root of the second molar

No significant difference in distance from the implantation site to the mandibular neural tube was observed for L6_mb_–L6_db_ and L7_mb_–L7_db_ regions (*P* > 0.05) (Table 3). The distances from the implantation site to the mandibular neural tube were all > 13 mm at planes of 3, 5, 7, and 9 mm for L6 and L7 regions.

**Table 3:** Distance from the implantation site to the mandibular neural tube (in millimeters: mean ± SD) at the planes of 3, 5, 7, and 9 mm

	L6_mb_–L6_db_		L7_mb_–L7_db_	
	*Х*	SD	*Х*	SD
3 mm	18.73	2.51	18.34	2.21
5 mm	16.27	1.88	16.29	2.13
7 mm	14.78	1.78	14.98	2.16
9 mm	13.40	1.70	13.72	2.19

*L6* central coronal section of the first molar, *L7* central coronal section of the second molar

## Discussion

The use of miniscrew has grown in popularity over the years because of its ability to provide absolute anchorage [10]. Distalization of the entire mandibular dentition is a viable way to correct a class III anteroposterior relationship (a negative overjet or an edge-to-edge occlusion) with miniscrews implanted in the MBS and dental and skeletal discrepancies of many patients could get dento-alveolar compensation [6]. However, the success of the method is closely related to the anatomical structures of the MBS. It is also critical to select an appropriate site in the MBS for miniscrew implantation. This site should provide good stability for the distalization of mandibular dentition without affecting the distal movement of teeth and the overall periodontal health. Although several investigators have evaluated cortical bone thickness and bone width by CBCT [5, 11, 12], a three-dimensional evaluation of safe region of miniscrew insertion in the MBS for mandibular dentition distalization has never been performed.

The MBS provides sufficient bone, thereby allowing miniscrew insertion on the buccal side of the tooth root and avoiding screw to root contact during distalization [13]. In this study, the width of the buccal alveolar in the posterior tooth area was significantly thicker than that in the anterior tooth area; therefore, the L6_db_–L7_mb_ and L7_mb_–L7_db_ regions were safer than the L6_mb_–L6_db_ and L5_db_–L6_mb_ regions for miniscrew insertion. To obtain sufficient orthodontic loading and stability, the length of the miniscrew biting depth in the bone should be at least 6 mm when the entire mandibular dentition was distalizated [14]. The narrowest buccal inter-radicular space in the L7_mb_–L7_db_ region was significantly lower than that of the L6_db_–L7_mb_ region at all planes. Considering the risk of miniscrew and root contact, the L6_db_–L7_mb_ region is the most reasonable and safest for miniscrew implantation in the MBS for the distalization of the entire mandibular dentition.

Although no signs or symptoms caused by root contact with miniscrews during mesiodistal movement of the molar have been reported, screw and root contact during insertion of miniscrews for orthodontic anchorage would increase the failure rate [13, 15]. In addition, a sufficient buccal bone mass and a large screw-root distance will further improve the success rate of miniscrews. Our results show that the mean values of the buccal alveolar bone and the inter-radicular space in the L6_db_–L7_mb_ region were 6.39 mm and 4.97 mm at a plane of 9 mm, respectively, with wider measurements at planes < 9 mm. As a minimum of 1 mm of alveolar bone around the screw is sufficient for good periodontal health [10], and considering that the diameters of most miniscrews are 1.2 to 2.3 mm [10], we conclude that it is safe for miniscrews to be inserted in the L6_db_–L7_mb_ region at and below a plane of 9 mm.

Miniscrew selection depends on multiple factors, including the insertion site, miniscrew material, cortical bone thickness, inter-radicular space, and method of miniscrew insertion [16]. On the one hand, the cortical bone thickness of the MBS was significantly greater than that of the other insertion sites. The anatomical features and loading force for the distilization of mandibular dentition indicated that miniscrews of greater sizes should be inserted in the buccal shelf. Therefore, we recommend using miniscrews 2 mm in diameter for implantation in the MBS to avoid excessive insertion torque and eventual fracture. On the other hand, systematic review revealed that the miniscrew length was closely related to the stability and success rate of the miniscrew. Crismani et al. showed that 8-mm miniscrews were associated with a 22% higher success rate than 6-mm ones [17]. In addition, the miniscrew length should be based on the location of adjacent anatomical structures (dental roots, nerves, and blood vessels) as well as the available bone depth. To avoid injury to vital anatomical structures during implantation, we evaluated the relationship between the inferior alveolar nerve and the implantation site. Our results showed that the distances from the implantation site to the mandibular neural tube were > 13 mm at a plane of 9 mm. Considering other factors, we recommend using 10-mm or 12-mm miniscrews in the L6_db_–L7_mb_ region at a plane of 9 mm. Of course, the miniscrew length should be shorter for smaller insertion sites.

Previous studies have reported the most favorable overall anatomic relationship for MBS orthodontic miniscrew placement to be at the level of the distobuccal cusp of the second molar, whereas the buccal bone and the cortical bone were the thickest in the MBS region [12, 18]. Due to limitations caused by the non-wide opening of patients’ mouths, the distal location of the insertion site can cause difficulties when accessing the site at the best angle. Furthermore, the thicker soft tissue can increase the failure rate of miniscrews [9]. Therefore, we still consider the L6_db_–L7_mb_ region as the best site in the MBS for the distalization of mandibular dentition.

Due to the limitation in the number of samples, many factors that might affect the measurement of the MBS, such as ethnicity, vertical skeletal patterns, and gender, were not considered [19]. Moreover, the thickness and morphology of the soft tissue in the MBS should also be studied, because the mobility of the alveolar mucosa can affect long-term miniscrew stability [10]. As such, further experiments will be needed to confirm our findings.

## Conclusion

According to an analysis of CBCT images, the region between the mandibular first and second molars (L6_db_–L7_mb_) should be the first choice for minisrew implantation in the buccal alveolar bone in the MBS for the distalization of the entire mandibular dentition.

## Data Availability

The data used to support the findings of this study are included within the article. The original data of each patient will also be used for research of other subjects. It is not convenient to release the data until the publication of the article. If necessary, the corresponding author can be asked for.

## Abbreviations

L5_b_: Buccal root of the mandibular second premolar
L6_db_: Distobuccal root of the mandibular first molar
L6_mb_: Mesiobuccal root of the mandibular first molar
L7_db_: Distobuccal root of the mandibular second molar
L7_mb_: Mesiobuccal root of the mandibular second molar
MBS: Mandibular buccal shelf
